# Female Chemical Signalling Underlying Reproduction in Mammals

**DOI:** 10.1007/s10886-018-0981-x

**Published:** 2018-07-11

**Authors:** Holly A. Coombes, Paula Stockley, Jane L. Hurst

**Affiliations:** Mammalian Behaviour and Evolution Group, Institute of Integrative Biology, University of Liverpool, Leahurst Campus, Chester High Road, Neston, CH64 7TE UK

**Keywords:** Chemical communication, Females, Sexual selection, Mammalian reproduction. pheromones

## Abstract

Chemical communication plays many key roles in mammalian reproduction, although attention has focused particularly on male scent signalling. Here, we review evidence that female chemical signals also play important roles in sexual attraction, in mediating reproductive competition and cooperation between females, and in maternal care, all central to female reproductive success. Female odours function not only to advertise sexual receptivity and location, they can also have important physiological priming effects on male development and sperm production. However, the extent to which female scents are used to assess the quality of females as potential mates has received little attention. Female investment in scent signalling is strongly influenced by the social structure and breeding system of the species. Although investment is typically male-biased, high competition between females can lead to a reversed pattern of female- biased investment. As among males, scent marking and counter-marking are often used to advertise territory defence and high social rank. Female odours have been implicated in the reproductive suppression of young or subordinate females across a range of social systems, with females of lower competitive ability potentially benefiting by delaying reproduction until conditions are more favourable. Further, the ability to recognise individuals, group members and kin through scent underpins group cohesion and cooperation in many social species, as well as playing an important role in mother-offspring recognition. However, despite the diversity of female scent signals, chemical communication in female mammals remains relatively understudied and poorly understood. We highlight several key areas of future research that are worthy of further investigation.

## Introduction

A defining feature of all mammalian taxa is high female investment in reproduction through the process of lactation (Wade and Schneider 1992). Gestation, which occurs in all species except monotremes, further increases female investment, as does parental care, which is provided solely by females in around 90% of mammalian species (Royle et al. 2014; Wade and Schneider 1992; West and Capellini 2016). The high costs of gestation and lactation mean that female reproduction is often constrained by food availability (Gittleman and Thompson 1988; Wade and Schneider 1992; West and Capellini 2016), resulting in female competition for access to food sources as well as other limiting resources such as space or shelter (Clutton-Brock 2009; Stockley and Bro-Jorgensen 2011). In addition to securing enough resources to successfully raise their offspring, females also need to ensure they mate with high quality males (Andersson 1994). This can benefit females through access to male resources, such as parental care or territory, and / or by gaining genetic benefits for their offspring (Andersson 1994; Clutton-Brock 2016; Kokko et al. 2003; Møller and Thornhill 1998). The importance of male quality to female reproduction has led to a focus on male signals used to attract females or to mediate competition between rival males (Clutton-Brock and McAuliffe 2009; Maynard Smith and Harper 2003; Wong and Candolin 2005). However, there is growing understanding that females also use signals for sexual attraction, to mediate female competition and cooperation, and to facilitate maternal behaviours (Clutton-Brock and Huchard 2013; Nowak et al. 2000; Stockley and Bro-Jorgensen 2011; Stockley et al. 2013).

Female chemical signals provide information on species, sex and individual identity, as well as current reproductive state, social status and health (Blaustein 1981; Brown and Macdonald 1985; Thiessen and Rice 1976). Such signals can function to attract males, advertise territory ownership or social rank, and to facilitate mate, group and offspring recognition (Heymann 2006; Nowak et al. 2000; Stockley et al. 2013). However, few female chemical signals have been identified as yet (Table 1), perhaps because of the focus on male signalling (see Liberles 2014 for a review of mammalian chemical signalling). In some species, females may produce more specialised signals known as pheromones. These are chemical signals used to communicate between members of the same species that trigger a specific behavioural response or physiological process (Karlson and Luscher 1959; Wyatt 2010). Female pheromones are recognised to induce sexual behaviours in males (Briand et al. 2004a) or to facilitating suckling behaviour in offspring (Schaal et al. 2003). Female mammals also use scents in a competitive context (Stockley et al. 2013), displaying similar strategies in the deployment of scent to those previously described in males (Gosling and Roberts 2001; Hurst and Beynon 2004). A common form of female competitive signalling is through scent marking (specialized motor patterns used to deposit chemical secretions on environmental objects or conspecifics (Ralls 1971)), which females use to advertise territory ownership and dominance rank (Stockley et al. 2013). Female scent marks can also advertise the current or approaching receptivity of the owner, or a female’s quality to potential mates (Blaustein 1981; Johnson 1973; Stockley et al. 2013). Once deposited, scent marks can be used by other animals to gain information about specific individuals, social groups or the areas they occupy, influencing future interactions and decisions (Brown and Macdonald 1985; Halpin 1986).

**Table 1:** Chemical compounds produced by female mammals^a^

**Chemical name**	**Species**	**Source and expression**	**Behavioural response**	**References**
(Z)-7-dodecen-1-yl acetate	*Elephas maximus*	Female urine, output peaks just prior to ovulation	Male flehmen, erections & premating behaviour	Rasmussen et al. (1997) Rasmussen et al. (1996) Rasmussen (2001) Rasmussen et al. (2005)
2-methylbut-2-enal	*Oryctolagus cuniculus*	Mammary glands of lactating females	Suckling behaviour and odour induced learning in pups.	Schaal et al. (2003)
Aphrodisin (odorant binding protein)	*Mesocricetus auratus*	Female vaginal secretions, output increases at oestrus	Male copulatory behaviour	Singer et al. (1986) Briand et al. (2004a)
2,5-dimethylpyrazine	*Mus musculus*	Adrenal glands of group housed non-breeding females, secreted in urine	Lengthening of oestrus cycle in females. Puberty delay of both sexes.	Jemiolo and Novotny (1994) Ma et al. (1998)
Major urinary proteins (MUPs)	*Mus musculus*	Liver, secreted in urine. Higher levels in males but output increases in females under competitive breeding conditions	Individual and kin recognition Status signalling in both sexes	Hurst et al. (2001) Green et al. (2015) Stockley et al. (2013)
Sulphated steroids	*Mus musculus*	Urine of adult females	Firing of vomeronasal sensory neurons in both sexes	Nodari et al. (2008) Hsu et al. (2008)
1-ido-2-methylundecane	*Mus musculus*	Urine of proestrus and oestrus females	Increases investigation and attraction to urine by males, though not as attractive as intact oestrus urine.	Achiraman et al. (2010)

^a^This table excludes volatile compounds that change across the female reproductive cycle, where a specific function has yet to be fully established.

Most studies investigating chemical communication in mammals have focused on male signalling (Apps 2013; Gosling and Roberts 2001; Hurst 2009; Johnson 1973), probably due to male-biased sexual dimorphism in both scent marking behaviour and glandular morphology found in many species (Blaustein 1981). However, female chemical signals are also important in mammalian reproduction, and in some species female investment equals or even exceeds that of males (Ferkin 1999; Heymann 1998; Sliwa and Richardson 1998). Male-biased dimorphism is often reduced in monogamous species while female-biased dimorphism occurs in species with high levels of female competition (Heymann 2006; Kleiman 1977). The investment in chemical signalling and deployment of scents by females is strongly influenced by the social structure and breeding system of the species, as well as the current physical and social environment of the signalling individuals. Here, we review current literature on chemical signals in female reproduction, with an emphasis on their role in sexual attraction, intrasexual competition and cooperation, and maternal behaviours.

## Sexual Attraction

Olfaction plays several key roles in mediating sexual and mating behaviours in mammals. As well as helping animals to locate and ensure appropriate recognition of opposite sex conspecifics, scents are also used as indicators of mate quality and for individual mate assessment (Johansson and Jones 2007). Further, chemical signals can be used to coordinate mammalian reproduction by altering the behaviour and physiology of both sexes (reviewed in Petrulis 2013). While the majority of studies have focused on males signalling to females (see Apps 2013; Burger 2005; Gosling and Roberts 2001; Roberts et al. 2014 for reviews), there is growing evidence that chemical cues are also produced by females to attract and stimulate males.

### Reproductive Advertisement

A widely studied function of female chemical signalling is to advertise sexual receptivity and fertility. Female scent marking often occurs at an increased frequency or exclusively during periods of sexual receptivity (rat: Birke 1978; meadow vole: Ferkin et al. 2004; coyote: Gese and Ruff 1997; domestic rabbit: Gonzalez-Mariscal et al. 1990; Hudson and Vodermayer 1992; general review: Johnson 1973; ringtailed lemur: Kappeler 1998; Canidae: Kleiman 1966; giant panda: Nie et al. 2012; klipspringer: Roberts and Dunbar 2000). Increased marking during these periods probably functions to attract mates, and females typically increase scent marking rates in the presence of male odours (meadow vole: Ferkin et al. 2004; tree shew: Holst and Eichmann 1998; domestic rabbit: Hudson and Vodermayer 1992; golden hamster: Johnston 1977). Advertisement of receptivity may be particularly important in solitary animals that need to attract mates from a distance and encounter conspecifics less frequently than more gregarious species (Waser and Jones 1983). In several solitary species, female marking rates peak just prior to oestrus, which may advertise their approaching receptivity and ensure a male is present during oestrus (golden hamster: Johnston 1977; tiger: Smith et al. 1989). Sexual advertisement can also be important in social or pair living species, where females deposit marks at territory edges to attract neighbouring males (aardwolf: Sliwa and Richardson 1998; yellow mongoose: Wenhold and Rasa 1994). Females may signal receptivity to increase competition between males, thereby increasing the likelihood of mating with males of high quality (Fischer and Brown 1993; Rasmussen et al. 1997). Males may gain a range of different information about a female’s reproductive state from various scent sources, e.g. saliva, urine, body glands and genital secretions, which combined may provide precise information about a female’s reproductive state (Lai et al. 1996).

The ability to distinguish between odours produced at different points in the female reproductive cycle is shared by many species, including domestic dog (Beach and Gilmore 1949), golden hamster (Huck et al. 1989), domestic sheep (Blissitt et al. 1990), cotton top tamarin (Ziegler et al. 1993), meadow vole (Ferkin and Johnston 1995), giant panda (Swaisgood et al. 2002) and ring-tailed lemur (Drea and Scordato 2008), with males often more attracted to female scents during periods of receptivity (e.g. domestic dog (Beach and Gilmore 1949), rat (Lydell and Doty 1972), golden hamster (Huck et al. 1989), giant panda (Swaisgood et al. 2002), meadow vole (Ferkin et al. 2004), cow (Sankar and Archunan 2004)). In strepsirrhine primates the volatile components of genital secretions vary between the breeding and non-breeding seasons (delBarco-Trillo et al. 2012; Drea and Scordato 2008; Greene and Drea 2014; Hayes et al. 2004; Morelli et al. 2013). Similar distinctions have been found in other species for urine (Barman et al. 2013; Vogt et al. 2016), faeces (Kimura 2001) and genital secretions (Harris et al. 2014). However, much of this work has been largely qualitative, with few studies identifying the compounds involved in discrimination or quantifying the level of change. Studies in several species have identified scent components that may be used to advertise female receptivity (mouse: Achiraman and Archunan 2006; Stopka et al. 2007; cow: Archunan and Kumar 2012; blackbuck: Archunan and Rajagopal 2013; elephant: Rasmussen et al. 1997). For example, in female Asian elephants (*Elephas maximus*) the urinary compound (*Z*)-7-dodencen-1-yl acetate elicits male flehmen responses, erections and premating behaviour (Rasmussen et al. 1996; Rasmussen et al. 1997). The concentration of (*Z*)-7-dodencen-1-yl acetate peaks just prior to oestrus, suggesting it functions to advertise the approach of female receptivity (Rasmussen 2001). Female receptivity may also be signalled by a combination of compounds. Studies in rats and bovine species found mixtures of compounds that change with female receptivity, with compound mixtures producing the strongest behavioural responses from males (Nielsen et al. 2011; Rajanarayanan and Archunan 2011; Sankar and Archunan 2008). A limitation, though, is that many of these studies focus on domesticated or laboratory animals and similar experiments on wild species are needed. While many of these compounds stimulate attraction and sexual behaviours in males (Achiraman and Archunan 2006; Achiraman et al. 2010; Archunan and Kumar 2012; Nielsen et al. 2011; Rajanarayanan and Archunan 2011; Rasmussen et al. 1997; Sankar and Archunan 2008), further tests are required to confirm their role in signalling female receptivity, including tests of response when female odours are specifically manipulated.

A particularly well studied example of female sexual advertisement concerns aphrodisin, a protein sex pheromone produced in the vaginal secretions of female golden hamsters (*Mesocricetus auratus*) that stimulates copulatory behaviour in males (Briand et al. 2004a; Singer and Macrides 1990). The expression of aphrodisin varies across the female reproductive cycle, reaching a maximum at oestrus (Briand et al. 2004a). Aphrodisin is an odorant binding protein that binds small hydrophobic molecules in vaginal secretions within its central cavity (Briand et al. 2000). The purified high molecular weight fraction of vaginal fluid containing aphrodisin stimulates mounting behaviour in male hamsters when detected through the vomeronasal system, even when painted onto anaesthetised males (Singer et al. 1986). This pheromonal effect may be due to low molecular weight ligands carried by aphrodisin, or to the complex of both protein and ligand, as recombinant aphrodisin alone was not effective in stimulating a copulatory response (Briand et al. 2004b).

Changes in female scent and scent marking behaviours are related to fluctuations in ovarian hormones across the female reproductive cycle (Takahashi 1990). Ovariectomy reduces or eliminates scent marking (golden hamster: Albers and Rowland 1989; domestic rabbit: Hudson and Vodermayer 1992; mouse: Kimura and Hagiwara 1985; Mongolian gerbil: Wallace et al. 1973), causes scent glands to regress (grasshopper mouse: Pinter 1985; Mongolian gerbil: Wallace et al. 1973) and reduces male attraction to female odours (meadow vole: Ferkin et al. 1991; domestic dog: Lisberg and Snowdon 2009). However, scent marking and glandular morphology can be restored through hormonal injections of estradiol (golden hamster: Albers and Rowland 1989; gray short-tailed opossum: Fadem 1990; mouse: Kimura and Hagiwara 1985) or estradiol and progesterone (rat: Birke 1984; Mongolian gerbil: Owen and Thiessen 1974). Hormone levels can also alter the chemical composition of female odours or the relative abundance of volatile chemicals in female scents (pine vole: Boyer et al. 1989; ringtailed lemur: Crawford et al. 2011; grey wolf: Raymer et al. 1986).

The best studied example of the hormonal control of female scent marking is vaginal marking in golden hamsters (reviewed in Been and Petrulis 2008). Vaginal marking peaks just prior to receptivity, but disappears during oestrus (Johnston 1977). This rise in vaginal marking is mediated by high levels of estradiol prior to receptivity (Lisk and Nachtigall 1988). The decline in marking during oestrus is probably due to falling levels of estradiol coupled with rising progesterone (Lisk and Nachtigall 1988). Implantations of estradiol and progesterone within the brain provided further evidence for the hormonal dependency of vaginal marking behaviour (Takahashi et al. 1985; Takahashi and Lisk 1985). However, vaginal marking rates also increase in the presence of male odours (Johnston 1977) and normal marking behaviour depends on an intact olfactory system (Johnston 1992; Petrulis et al. 1999), showing that vaginal marking is regulated by external chemosensory cues as well as internal hormonal cycles.

Have females evolved specific signals to inform males of their receptivity, or have males learned to detect changes in female cues that occur as a by-product of hormonally driven physiological changes? Although experimentally differentiating between signals and cues can be difficult, their evolutionary implications can be very different (Otte 1974). Signalling implies intentional information transfer which benefits the signaller, for example if females signal receptivity to attract a mate in solitary species or to gain benefits from mating with multiple males (Reynolds 1996; Steiger et al. 2010). However, when males detect changes in female cues that females are not actually signalling, this can have neutral or even detrimental effects on females (Otte 1974).

While advertisement of female receptivity has been widely investigated, the extent to which females signal outside of mating has received much less attention. Scent marking rates in female golden hamsters, a solitary species, are lowest during pregnancy and early lactation (Johnston 1979) and males are less attracted to odours from pregnant females (Johnston 1980). Reduced sexual advertisement during these periods could prevent males approaching pregnant or lactating females, reducing the risk of infanticide to their pups. Alternatively, the high energetic costs of gestation and lactation may limit female scent marking during this period (Clutton-Brock et al. 1989; Gubernick and Klopfer 1981; Wade and Schneider 1992). Male meadow voles (*Microtus pennsylvanicus*) are significantly less attracted to female odours immediately prior to parturition, although pre-parturition odours are still more attractive than male odours (Ferkin and Johnston 1995). Pregnant and lactating females are highly aggressive in many species (Svare 1981), so this decrease in male attraction may be due to females emitting a “stay away” signal. By advertising an increase in aggressiveness, females may prevent conspecifics from approaching them and their pups. Unfamiliar males and females pose a risk to newborn young in many species (Ebensperger 1998), so female “stay away” odours should result in avoidance by both sexes. However, the response of female voles to pre-parturition odours was not tested. By contrast, the odours of female voles in post-partum oestrus, a period of receptivity that occurs soon after parturition, are highly attractive to males (Ferkin and Johnston 1995). Females may advertise receptivity rather than aggression during postpartum oestrus, as the necessity to attract a mate may outweigh the desire to prevent conspecifics approaching vulnerable pups during this period. Alternatively, the attractiveness of female receptivity cues may cause males to ignore female signals of aggression.

Instead of advertising receptivity, some female mammals may try to conceal their reproductive state. In several socially monogamous species, where a single breeding male and single breeding female share a common range or territory and associate with each other for more than one breeding season (Lukas and Clutton-Brock 2013), female scent marking rates do not change across the reproductive cycle (pygmy marmoset: Converse et al. 1995; prairie vole: Wolff et al. 2002). Attracting males during periods of receptivity may be less important in monogamous species and concealed ovulation has been linked to the evolution of monogamy in primates (Alexander and Noonan 1979 but see Dixson 2012). However, lack of advertisement is not the same as concealment and socially monogamous males can still discriminate reproductive state from female odours (pygmy marmoset: Converse et al. 1995; cotton-top tamarin: Ziegler et al. 1993). Socially monogamous females can engage in extra-pair copulations so, may still benefit from advertising receptivity to neighbouring males (Birkhead and Moller 1992; Clutton-Brock and Isvaran 2006). Further, given the extent to which hormone levels can influence chemical secretions (see references above), and the sensitivity of vomeronasal receptors to sulphated derivatives of all major classes of steroid hormones excreted in urine (Nodari et al. 2008), it seems unlikely that females could ever completely conceal their reproductive state.

### Effects of Female Chemosignals on Male Physiology

While the priming effects of male chemosignals on female reproductive physiology have been well studied (Koyama 2004; Koyama 2016), potential effects of female chemosignals on males have received less attention (Petrulis 2013). Male laboratory mice housed with an unrelated adult female reach sexual maturity faster than when housed alone or with an unrelated adult male (Vandenbergh 1971), while exposure to female scents can increase testis and seminal vesicle weights in juvenile male rodents (Babb and Terman 1982; Purvis and Haynes 1972; Terman 1984; Wayne and Rissman 1990). Puberty acceleration in males following exposure to adult females may help coordinate reproduction between the sexes. Further, males may increase their reproductive success by reaching puberty earlier when sexually mature females are available, particularly in short-lived rodents where male mortality is high (Berry and Bronson 1992; Promislow and Harvey 1990; Triggs 1991).

Exposure to an unfamiliar female or her odours causes an increase in plasma testosterone in male mice (Macrides et al. 1975), rats (Bonilla-Jaime et al. 2006), hamsters (Macrides et al. 1974), marmosets (Ziegler et al. 2005), macaques (Cerda-Molina et al. 2006) and humans (Miller and Maner 2010; but see Roney and Simmons 2012). This increase in testosterone occurs 30-60 minutes after initial exposure (Cerda-Molina et al. 2006; Richardson et al. 2004; Ziegler et al. 2005) and is often preceded by an increase in circulating luteinizing hormone (Cerda-Molina et al. 2006; Richardson et al. 2004). Although the adaptive functions of these hormonal changes are currently unknown, high testosterone levels are linked to many male-specific traits, as well as male mating success, aggression and dominance rank (Beehner et al. 2006; Mills et al. 2009; Muller 2017; Setchell et al. 2008; Wickings and Dixson 1992). Many male chemosignals are androgen dependent (mice: Achiraman and Archunan 2005; Harvey et al. 1989; Novotny et al. 1985; goat: Iwata et al. 2000; domestic pig: Loebel et al. 2001; rat: Ponmanickam et al. 2010), so increased levels of male hormones may increase production of male chemical signals that are attractive to females (Roberts et al. 2010; Zhang et al. 2008). However, hormonal increases in male laboratory mice were not dependent upon female reproductive state (Maruniak and Bronson 1976). The components of female scents that elicit surges in male hormones are yet to be identified, but in mice low molecular weight molecules bound by major urinary proteins (MUPs) may be responsible (Singer et al. 1988).

Testosterone is essential for spermatogenesis (Smith and Walker 2014; Walker 2011), so the elevation in testosterone in response to female chemical signals may function to increase sperm production. In agreement with this, increased sperm production in response to female chemical signals has been reported in laboratory rodents (Koyama and Kamimura 2000; Taylor et al. 1987). Dominant male laboratory mice, but not subordinates, show increased sperm density when housed with female bedding (Koyama and Kamimura 2000) and odour from group housed females produces a greater increase than odours from isolated females (Koyama 2004). However, a study in wild house mice (*Mus musculus*) found that although males showed plasticity in sperm production, this was caused by competitive cues from other males, rather than by female cues relating to mating opportunities (Ramm et al. 2015).

Male hormonal responses to females are context dependent, as male rodents quickly habituate to presentation of the same female, and the greatest elevation of luteinising hormone and testosterone follows exposure to a novel female (Coquelin and Bronson 1979; Shulman and Spritzer 2014). Further, hormone surges can be linked to neutral olfactory cues through learned association with receptive females (Graham and Desjardins 1980). Social conditions impact endocrine response in male common marmosets (*Callithrix jacchus*): males housed alone or with a female show elevated testosterone following exposure to a novel female odour but males housed in family groups do not (Ziegler et al. 2005). Male marmosets contribute to parental care but elevated levels of testosterone are linked to male aggression (Dixson 1980; Honess and Marin 2006; Rose et al. 1971). Those in family groups may inhibit the normal elevation of testosterone to minimise aggressive behaviours towards vulnerable infants. However, during control tests, males housed in families or pairs tended to have slightly higher testosterone levels than singly housed males, although the difference was not statistically significant (Ziegler et al. 2005). Alternatively, elevation of testosterone in response to female scents may function to increase male mating rates, for example through increasing spermatogenesis or production of attractive male chemosignals, which may be less important in socially monogamous male marmosets housed in a stable family group.

The renewal of sexual behaviour following mating when males are exposed to a novel female, called the Coolidge effect, occurs in many male mammals, including rats (Bermant et al. 1968; Brown 1974; Wilson et al. 1963), voles (Dewsbury 1973; Gray and Dewsbury 1975), cats (Whalen 1963), sheep (Pepelko and Clegg 1965) and hamsters (Bradford et al. 1977). Males often show more chemosensory investigation of novel females, suggesting that chemical cues may allow males to recognise familiar females (Johnston and Rasmussen 1984). Consistent with this, disruption of the main olfactory system abolishes the preference of sexually satiated male golden hamsters for a novel female (Johnston and Rasmussen 1984). Recent studies in laboratory rats have shown that sexually satiated males fail to ejaculate semen, as no spermatozoa or seminal plugs are found in the female genital tract following copulation. Further, sexually satiated male rats impregnate significantly fewer females than do rested males (Lucio et al. 2014). An alternative function of the Coolidge effect may be to reduce the probability of fertilization by rival males. In laboratory rats, sexually satiated males dislodge seminal plugs deposited by previous males, reducing sperm from rival males in the female’s genital tract (Lucio et al. 2014). Notably, no female rats that had recently mated gave birth after copulating with another male under the Coolidge effect (Lucio et al. 2014). The absence of any renewal of sexual behaviour in socially monogamous rodents on exposure to a novel female (Dewsbury 1971; Pierce et al. 1992) provides further evidence that the Coolidge effect may function to mediate post-copulatory competition between males in promiscuous species. Mating with multiple males can increase female reproductive fitness (Jennions and Petrie 2000), so females may not necessarily benefit from mating under the Coolidge effect.

### Female Signals of Quality

While females often prefer males that deposit the greatest number of scent marks, and high rates of scent marking among males have been linked to their quality and competitive ability (Gosling and Roberts 2001; Rich and Hurst 1999), the extent to which female chemosignals are used by males to select a mate of high quality has received little attention. Further, the influence of female scent marking rate on male mate choice remains largely unknown. Females of several species, including bush dogs (Porton 1983), cotton top tamarins (French and Cleveland 1984) and moustached tamarins (Heymann 1998), display more frequent and diverse marking behaviour than males, and males typically spend more time investigating female scents (Heymann 1998). Female-biased marking in these species is probably due to high levels of male parental care, leading to increased female competition and male mate choice. Among callitrichid primates, scent marking rates are female-biased in species with male-biased parental care, but male-biased when parental care is equal or greater in females (Heymann 2006). The increased cost of marking to females may be offset by the assistance in offspring care from males.

Female preference between male scents can depend upon male status (Kruczek 1997; Mossman and Drickamer 1996; Zhang et al. 2001), health (Kavaliers and Colwell 1995; Willis and Poulin 2000) and genetic quality of the scent owner (Ilmonen et al. 2009; Thom et al. 2008). Few studies have investigated whether similar factors influence male preferences between female scents. Male ring-tailed lemurs (*Lemur catta*) are more attracted to scents from dominant females, but only if scents are from familiar females (Scordato and Drea 2007). The few studies that have investigated female quality signalling and male preference have focused on species in which females are dominant. Studies in species covering a range of other social systems are also needed.

Whether female chemical signals produce honest signals of quality has rarely been investigated, probably because female signalling is expected to be limited by the high costs of offspring production (Chenoweth et al. 2006; Fitzpatrick et al. 1995; Nordeide et al. 2013). Ferkin et al. (1997) found that male meadow voles were more attracted to odours from females fed on a high, compared to a low, protein diet. Female meadow voles occupy exclusive territories in areas where food patches often differ in quality (Bowers et al. 1996; Madison and McShea 1987). A protein-rich diet may provide a good indicator of a female’s ability to hold a high quality territory successfully, so may be a true indicator of female quality or ability to invest in offspring. In ring-tailed lemurs, individual heterozygosity correlates negatively with diversity of fatty acids and positively with the diversity of heavy fatty acid esters (Boulet et al. 2010). The authors argue that this may be an example of honest olfactory signalling as genetic heterozygosity correlates with health and survivorship in this captive population (Charpentier et al. 2008). However, subsequent behavioural tests revealed that males tended to spend more time near scent from less heterozygous females not less time, although this difference was not statistically significant (Charpentier et al. 2010). Chemical diversity correlates positively with genetic heterozygosity in female Antarctic fur seals (*Arctocephalus gazella)* (Stoffel et al. 2015). As heterozygosity increases early survivorship and breeding success in female fur seals (Stoffel et al. 2015), greater chemical diversity could be an indicator of female quality. However, behavioural tests are needed to test whether males prefer more heterozygous females and their scents.

## Intrasexual Competition and Cooperation

Scent signals can play an integral role in mediating both competitive and cooperative interactions between females (Stockley et al. 2013). The high cost of lactation and gestation means that females frequently compete for access to resources (Stockley and Bro-Jorgensen 2011), using scent marks to signal ownership of particular resources or areas containing resources (Kruuk 1992; Miller et al. 2003; Ralls 1971). In group living species odours can be used to signal social rank (Heymann 2006; Ralls 1971), with high ranking females often benefiting from priority access to resources and high quality mates (Côté and Festa-Bianchet 2001; Pusey et al. 1997; van Noordwijk and van Schaik 1999). Females often counter-mark the scents of other females, by depositing scent marks on top of or adjacent to the original mark (Ewer 1968), to signal competitive ability (Gosling 1982; Rich and Hurst 1999). Odours also allow recognition of group members or cooperative partners, which may be particularly important in species that rear their young together, providing benefits through group defence as well as cooperative hunting, nesting and / or nursing (Gittleman 1989; Jennions and Macdonald 1994; Packer et al. 1990). Chemical signals have been linked to reproductive suppression in cooperatively breeding species, where reproduction is monopolised by the dominant pair within a group and subordinate females assist with offspring care (Clutton-Brock et al. 2001; Creel et al. 1997; Faulkes and Bennett 2001; French 1997). As discussed below, levels of female cooperation or competition depend on the degree of reproductive synchronisation, the social structure and mating system of the species, the relative ages of the competing females and current environmental conditions.

### Territorial Marking

Female reproductive success is often constrained by the availability of resources such as food, water or shelter (Clutton-Brock 2009; Stockley and Bro-Jorgensen 2011). To maintain access to limiting resources, females often establish territories that they defend either alone or as part of a group (Ostfeld 1985; Stockley and Bro-Jorgensen 2011). Scent marking is used to advertise territory ownership in many species (e.g. common vole: Dobly 2005; general review: Gosling 1982; spotted hyena: Henschel and Skinner 1991; banded mongoose: Müller and Manser 2008; European rabbit: Mykytowycz 1965; aardwolf: Richardson 1991; klipspringer: Roberts and Dunbar 2000; Eurasian beaver: Rosell et al. 1998; Ethiopian wolf: Sillero-Zubiri and Macdonald 1998; tiger: Smith et al. 1989). The presence of foreign scent marks induces investigation from the territory owner (Palagi et al. 2005; Sliwa and Richardson 1998) followed by an increased rate of scent marking, particularly when intruders are the same sex (Dobly 2005; Hurst 1990; Johnston 1977; Sliwa and Richardson 1998). Territorial marking may be an honest form of signalling as only successful territory holders will have the most abundant and / or overall freshest scent marks, providing a continuous record of ownership (Gosling and Roberts 2001; Rich and Hurst 1998).

Territorial marking can also influence the spacing behaviour of mammals. In honey badgers (*Mellivora capensis*) token urination is performed almost exclusively by females and patterns of marking do not vary across seasons, suggesting it may function to maintain spatiotemporal separation between females (Begg et al. 2003). Scents may also effect spacing behaviour in golden hamsters as female hamsters avoid areas marked by other females (Fischer and McQuiston 1991). As resident animals are more likely to defend their home territory, the avoidance of areas scent marked by conspecifics may reduce costly aggressive encounters between females (Roberts 2012). Further, female golden hamsters are more likely to attack an intruder when their own odour is present in the local environment and the presence of a previously subordinate female’s scent can cause a reversal in dominance (Fischer and McQuiston 1991). While territorial marking does not directly prevent other individuals from entering a territory, it does allows intruders to make an informed decision about the relative costs or benefits before entering (Gosling 1982). Females may use the chemical composition of scent marks to assess the competitive ability of territory holders. Female house mice increase investment in MUPs when faced with competition from neighbouring females (Garratt et al. 2011). The frequency of aggressive behaviours towards unfamiliar females is also strongly related to the urinary protein output of the aggressor, with more aggressive females exhibiting higher protein investment (Stockley et al. 2013). Under high densities, female reproductive success is strongly influenced by the ability to successfully defend a territory (Hurst 1987), so investment in urinary protein in scent marks may deter intruding females by serving as an indicator of investment in territory defence by the resident female.

Territorial animals often differ in their behaviour towards familiar neighbours compared to unfamiliar animals. Many mammals can discriminate between odour from neighbouring individuals and strangers (giant kangaroo rat: Murdock and Randall 2001; European badger: Palphramand and White 2007; Eurasian beaver: Rosell and Bjørkøyli 2002; aardwolf: Sliwa and Richardson 1998) and typically spend longer investigating scents from unknown conspecifics (African lion: Gilfillan et al. 2017; European rabbit: Monclús et al. 2014; Colombian ground squirrel: Raynaud and Dobson 2011; Eurasian beaver: Rosell and Bjørkøyli 2002). When presented with translocated scent marks from neighbouring conspecifics, aardwolves (*Proteles cristata*) immediately visit and scent mark their shared border, suggesting that they are capable of recognising neighbouring individuals (Sliwa and Richardson 1998). Territory holders may reduce the energetic costs of territorial defence by decreasing aggression towards neighbouring conspecifics, the so called the “dear enemy phenomenon” (Fischer 1954; Temeles 1994; Ydenberg et al. 1988). Female meadow voles display less antagonistic behaviour towards females with a familiar scent they have previously encountered (Ferkin 1988). Additionally, female bank voles (*Myodes glareolus*) display higher rates of infanticide towards the offspring of unfamiliar females under semi-natural conditions (Ylonen et al. 1997). However, European rabbits (*Oryctolagus cuniculus*) exposed to repeated simulation of intrusions by neighbours increase their rates of counter marking, suggesting that animals adapt their behaviour to neighbours depending upon the perceived level of threat (Monclús et al. 2014).

The “nasty neighbour” hypothesis predicts that, rather than reducing agonistic behaviour, residents will display increased levels of aggression towards neighbouring conspecifics (Müller and Manser 2007). Banded mongoose groups (*Mungos mungo*) emit more worry calls and perform more inspection bouts in response to translocated scent marks from neighbouring groups compared to the scent marks of strangers (Müller and Manser 2007). Social animals often disperse alone or in small groups, so strangers may pose less threat to a group’s resources than neighbouring groups of similar size (Cant et al. 2001; Cant et al. 2002). Intolerance of neighbours may also increase with population density. Female mound building mice (*Mus spicilegus*) tend to be more aggressive towards their immediate neighbours, particularly when pregnant (Simeonovska-Nikolova 2012). Although the sex ratio is equal at the beginning of the breeding season, it becomes female biased during the late summer (Simeonovska-Nikolova 2012). The declining nutritional value of food resources coupled with the rising density of females during summer months increases competition between neighbours (Simeonovska-Nikolova 2012).

### Dominance Signals

Scent signals are used to advertise and maintain social or reproductive dominance across a wide range of species (Barrette 1977; Ralls 1971). Dominant females often display higher rates of scent marking (Callitrichidae: Epple 1972; coyote: Gese and Ruff 1997; golden hamster: Johnston 1977; meerkat: Jordan 2007; African wild dog: Jordan et al. 2013) and counter-marking (banded mongoose: Müller and Manser 2008; ringtailed lemur: Palagi et al. 2004), and may have larger and more complex scent glands (golden hamster: Drickamer and Vandenbergh 1973). In ring-tailed lemurs, high-ranking females counter-mark the genital marks of other females more frequently than low-ranking females do (Palagi et al. 2004). Similarly, dominant female banded mongooses counter-mark the scents of other females at higher rates than subordinates (Müller and Manser 2008; but see Jordan et al. 2011). Rates of counter-marking by female mongooses increase during oestrus, suggesting that counter-marking may be involved in female competition for males during the breeding season. Further, in *Eulemur*, the group of Lemuridae known as brown lemurs, the chemical complexity of genital secretions is male-biased in subspecies that lack rank relations between the sexes (co-dominated social structure) but female-biased in female dominated species (delBarco-Trillo et al. 2012). However, as the chemical complexity of female secretions does not differ between co-dominated and female dominated species (delBarco-Trillo et al. 2012), this female-biased chemical complexity is probably due to a reduction in male signal complexity in female dominated species, rather than a greater complexity in females.

Different patterns of scent marking rates in relation to female social rank have also been reported. In common marmosets, subordinate females scent mark more frequently than dominant females during intergroup encounters (Lazaro-Perea et al. 1999). Similarly, subordinate female yellow mongooses (*Cynictis penicillata*) deposit more scent marks than both dominant and juvenile females and concentrate most scent marking at territory borders (Wenhold and Rasa 1994). Subordinate females often visit neighbouring territories during oestrus and are mated by neighbouring males (Wenhold and Rasa 1994), suggesting that their marking functions to advertise their presence to potential mates. Interestingly, a subsequent study carried out in a different population of yellow mongoose found no increased scent marking rates by subordinate females at territory borders (Le Roux et al. 2008). The difference between these two studies may be due to a difference in population density. Le Roux et al. (2008) studied a low-density population where females dispersed into new territories upon reaching sexual maturity. At the higher densities studied by Wenhold and Rasa (1994), females may have been unable to disperse so instead searched for mating opportunities within neighbouring groups.

Patterns of female scent marking also depend on whether females compete for reproductive opportunities within their group or between neighbouring groups. Alpha female golden lion tamarins (*Leontopithecus rosalia*) only display higher rates of scent marking than subordinates during intergroup encounters (Miller et al. 2003). Subordinate females are often daughters of the alpha pair, so pose little threat to the dominant female’s reproduction (Miller et al. 2003). Dispersing females can only enter a group as the alpha female (Baker and Dietz 1996), therefore alpha females may increase scent marking rates in the presence of female intruders to advertise their presence and deter other females from attempting to immigrate (Miller et al. 2003).

### Reproductive Suppression and Synchronisation

Synchronisation of oestrus has been reported in a number of mammalian taxa, particularly among primates and rodents (French and Stribley 1987; Handelmann et al. 1980; McClintock 1971; McClintock 1978; Wallis 1985; Weller and Weller 1993; Weller and Weller 1997). Ovarian synchronisation may reduce monopolization of females by dominant males, allowing females to pursue copulations with other mates (Emlen and Oring 1977). Early studies in laboratory rats suggested this synchronisation was mediated by female chemical signals (McClintock 1978; McClintock and Adler 1978; McClintock 1984). Similarly, the timing of ovulation was suggested to be under female pheromonal control in humans (Preti et al. 1986; Russell et al. 1980; Stern and McClintock 1998). However, many of these original studies have been criticized for methodological and statistical errors (Arden and Dye 1998; Schank 2000; Schank 2001a; Strassmann 1999; Wilson 1987; Wilson 1992) and subsequent studies found no evidence for synchronisation (Erb et al. 1993; Fürtbauer et al. 2011; Monfort et al. 1996; Schank 2001b; Setchell et al. 2011; Strassmann 1997; Tobler et al. 2010; Trevathan et al. 1993; Yang and Schank 2006). Given the variability within and between female ovarian cycles, it is argued that true synchronisation of oestrus is highly unlikely because matching will not be achieved across multiple cycles (Schank 2000). Additionally, many of these studies investigated oestrus synchrony in captive animals, where breeding is artificially regulated, or in western human populations, where women often use contraceptives to control reproduction. Such studies may not be representative of natural populations where females spend significantly more time pregnant or lactating (Strassmann 1997).

As synchronisation of oestrus may lead to high levels of competition between females and to male mate choice, females may actually benefit by avoiding ovarian synchronisation (Emlen and Oring 1977; Schank 2004). Asynchronisation of oestrus has been reported in several species (golden hamster: Gattermann et al. 2002; chimpanzee: Matsumoto-Oda et al. 2007; ring-tailed lemur: Pereira 1991) and may function to reduce female competition for mates (Schank 2004). Alternatively, females may delay reproduction during periods of high competition. Oestrus cycles in group housed female laboratory mice are significantly longer than in mice housed alone or in small groups of 2-3, known as the Lee Boot effect (Champlin 1971; Whitten 1959). Replication of the Lee-Boot effect through exposure to urine from group housed anoestrus females indicates that this is mediated by olfactory cues (Drickamer 1974; McIntosh and Drickamer 1977). Subsequent tests revealed 2,5-dimethylpyrazine, produced by group-housed non-breeding females, to be the key component in lengthening the oestrus cycle (Ma et al. 1998; Novotny et al. 1986). The magnitude of the Lee-Boot effect correlates positively with both the density of non-breeding females and the length of time they are in groups (Coppola and Vandenbergh 1985). This suggests that it may function to prevent female reproduction at high population densities when pup survival is poor among crowded females (Christian and Lemunyan 1958; Southwick 1955a; Southwick 1955b). Delaying reproduction during periods of high competition may increase long term reproductive success in females of lower competitive ability (Wasser and Barash 1983).

Exposure to 2,5-dimethylpyrazine also causes puberty delay in both male and female mice (Jemiolo and Novotny 1993; Jemiolo and Novotny 1994). Urine from wild mice living at high density delays puberty in laboratory mice, providing a mechanism that can delay reproduction in natural populations under conditions of increased resource competition (Massey and Vandenbergh 1980). In support of the suppressive effects of 2,5-dimethylpyrazine, urine and soiled bedding from group housed females reduced population growth in mice living in two large outdoor enclosures due to a combination of fewer females attaining puberty and at a later age (Drickamer and Mikesic 1990). As reproductive success is often linked to body size and condition, young females particularly may benefit from delaying reproduction (McNamara and Houston 1996). Delayed sexual maturation has been reported in other female rodents (vole species: Batzli et al. 1977; Mongolian gerbil: Clark and Galef 2002; prairie vole: Getz et al. 1983; california mouse: Gubernick and Nordby 1992; deermouse: Haigh 1983; pine vole: Schadler 1990). However, the mechanisms of suppression in these species are yet to be determined, with some evidence for both behavioural (Brant et al. 1998; Gubernick and Nordby 1992) and chemical mechanisms (Batzli et al. 1977; Getz et al. 1983; Schadler 1990). Further, a study by Wolff et al. (2001) found no evidence that female meadow voles (*M. pennsylvanicus*) or prairie voles (*Microtus ochrogaster*) suppress reproduction in the presence of their mother. In some species, puberty delay may not be inhibited by female signals, but females may require the presence of an unfamiliar male to stimulate reproduction (Mongolian gerbil: Clark and Galef 2002; prarie vole: Hofmann and Getz 1988; McGuire and Getz 1991). Exposing young female Mongolian gerbils (*Meriones unguiculatus*) to an unfamiliar male accelerates development even in the presence of their reproductively active mother (Clark and Galef 2002). Stimulation by an unfamiliar male may reduce the risk of inbreeding among young females.

In many cooperatively breeding species, dominant females suppress subordinate reproduction (common marmoset: Abbott et al. 1988; Damaraland mole-rat: Bennett 1994; African wild dog: Creel et al. 1997; meerkat: O’Riain et al. 2000; general-review: Solomon and French 1997; Ethiopian wolf: van Kesteren et al. 2013). This benefits dominant females by reducing competition for resources, as well gaining assistance from suppressed subordinates in offspring care (Hodge 2009). Olfactory cues from dominant individuals have been implicated in the reproductive suppression of subordinate females in callitrichid primates (Barrett et al. 1990; Epple and Katz 1984; Heistermann et al. 1989; Savage et al. 1988). However, only odours from familiar, dominant females inhibit ovulation, suggesting that odours inhibit reproduction by signalling the presence of the familiar dominant rather than through a pheromonal cue produced by all dominant females (Abbott et al. 1997). Further, female common marmosets that remained in visual, but not in olfactory, contact with their dominant female were still reproductively suppressed, again indicating that suppression is not directly caused by a chemical cue (Barrett et al. 1993). Instead subordinate females may learn to associate cues from a familiar dominant female with the behavioural subordination that the female imposes.

If odour signals from dominant females function as a threat, reproductive suppression in subordinates may be self-imposed (Johnstone and Cant 1999; Saltzman et al. 2009). This may allow subordinates to avoid costly aggressive encounters including eviction from the group and infanticide (Clutton-Brock et al. 1998; Kutsukake and Clutton-Brock 2006; Saltzman et al. 2009; Young et al. 2006). Additionally, subordinate females may increase survival and long-term reproductive success by remaining on their natal territory until they can either replace the dominant female or disperse (Clutton-Brock et al. 1998; Clutton-Brock et al. 1999; Rood 1990). As subordinate females are often the offspring of the dominant pair, they may also gain indirect fitness benefits from assisting with raising siblings (Emlen 1995; Griffin and West 2003). In some species, self-imposed reproductive suppression may function to reduce inbreeding (Snowdon 1996). In Damaraland mole rats (*Cryptomys damarensis*) females continue to exhibit reproductive suppression 30 days after being removed from the dominant female or her cues (Clarke et al. 2001). Exposure to an unrelated male results in rapid onset of reproductive activation, even in the presence of the dominant female (Clarke et al. 2001; Cooney and Bennett 2000). Breeding by subordinate females following the introduction of an unrelated male has been reported in other cooperatively breeding species (common marmosets: Digby 1995; Saltzman et al. 2004; meerkats: O’Riain et al. 2000). However, subordinates still have significantly lower reproductive rates than dominant females (Digby 1995; O’Riain et al. 2000), suggesting that reproductive suppression may be caused by an interplay between rank-related breeding and inbreeding avoidance.

### Choice of Social Partners

Olfactory cues can promote group cohesion and cooperation in gregarious species. Group living individuals benefit from reduced predation and increased success at locating or maintaining access to resources (Silk 2007). Group members often scent mark at communal marking sites, which may facilitate information transfer within the group and encourage group cohesion (Gittleman and Thompson 1988; Johnson 1973; Porton 1983; Sillero-Zubiri and Macdonald 1998). In spotted hyenas (*Crocuta crocuta*) anal gland secretions from high ranking females are preferentially overmarked by subordinates (Burgener et al. 2009). During overmarking, individuals “anoint” themselves with scent from the previous donor, a behaviour that may be involved in advertising continued clan membership (Burgener et al. 2008). Female marking rates decline with age, with young adult females displaying the highest marking rates, indicating a greater need for younger females to advertise group membership (East et al. 2013). Advertisement of group membership may be particularly important in spotted hyenas, which live in fission-fusion groups, where females may be absent from the clan territory for several days on long distant foraging trips (Hofer and East 1993; Kruuk 1972).

Members of the same group may have a distinctive shared odour (big brown bat: Bloss et al. 2002; spotted heyenas: Burgener et al. 2008; European badger: Gorman et al. 1984; meerkat: Leclaire et al. 2017; Bechstein’s bat: Safi and Kerth 2003). In some species of bats, females can identify roost-mates by scent (Bloss et al. 2002; Bouchard 2001; De Fanis and Jones 1995) and will attack foreign females that enter the colony (Kerth et al. 2002). Additionally, the volatile components of odours from different colonies are chemically distinct in Bechstein bats (*Myotis bechsteinii,* (Safi and Kerth 2003) and big brown bats (*Eptesicus fuscus*, (Bloss et al. 2002). In both species, females raise their offspring in maternal colonies which, despite disintegrating over winter, are stable over time as females return to the same colony each year (Bloss et al. 2002; Kerth et al. 2002; Kerth et al. 2011). Further, within some colonies females display fission-fusion societies, where females split into subgroups to occupy different day roosts (Kerth and Barbara 1999; Kerth et al. 2011), suggesting that colony specific odours may facilitate long term group stability. Group specific odours have also been reported in hyenas (Burgener et al. 2008), meerkats (Leclaire et al. 2017) and badgers (Gorman et al. 1984) and may be caused by differences in bacterial communities between groups (Leclaire et al. 2014; Leclaire et al. 2017; Theis et al. 2012; Theis et al. 2013). The fermentation hypothesis predicts that bacteria within scent glands metabolize glandular secretions, producing compounds that are used by the host to communicate with conspecifics (Albone and Gronnerberg 1977; Archie and Theis 2011; Gorman 1976). Members of the same social group harbour more similar odour-producing bacteria in their scent glands than members of different social groups (Archie and Theis 2011). Using GC-MS and deep sequencing techniques, Leclaire et al. (2017) found that the chemical composition of anal gland secretion and the composition of bacterial communities within the anal gland varied with group membership in wild meerkats, *Suricata suricatta*. These group-specific bacterial communities could arise through cross-infection from allomarking or rapid overmarking within groups (Buesching et al. 2003; Burgener et al. 2008; Theis et al. 2008).

Choice of social partners may be particularly important in species that care for offspring communally. Female house mice often raise their young in a communal nest (Manning et al. 1995; Wilkinson and Baker 1988), in which dams combine all their offspring and share maternal duties including nursing (König 1989a). Although prior familiarity between partners is a major factor influencing the success of communal nests (König 1993; König 1994), nesting with closely related females may also be beneficial (König 1994). Odour similarity correlates with genetic similarity (referred to as odour-gene covariance) across a range of species, providing a mechanism for assessing genetic relatedness among conspecifics through scent (Boulet et al. 2009; Todrank and Heth 2003; Tzur et al. 2009). However, the influence of many non-genetic factors on scents reduces the reliability of assessing degree of relatedness simply from overall chemical similarity (Hurst and Beynon 2010). While female house mice prefer unfamiliar nestmates that are related over equivalently unrelated females, their strongest preference is for nestmates that share the same polymorphic pattern of MUPs in their urine, which they can detect through urine scent (Green et al. 2015). As only females that are very closely related are likely to express the same inherited pattern of MUPs, this will be a highly reliable marker of close relatedness between females. Reliable assessment of close relatedness may be particularly important in the context of communal nursing when potential partners for cooperation are not highly familiar littermate sisters, allowing females to gain indirect fitness benefits from lactation investment in offspring that are not their own.

## Maternal Behaviours

In mammals, the cost of reproduction continues after birth as neonates are entirely dependent upon their mother (Gubernick and Klopfer 1981). Postnatal investment represents a significant cost to females and reduces their opportunity to produce additional offspring, so there should be strong selection to ensure that own offspring are the beneficiaries (Clutton-Brock 1991). Chemical cues can be used for both offspring recognition and maternal recognition (Nowak et al. 2000) and odours facilitate suckling behaviour in a number of mammals (Arteaga et al. 2013; Schaal and Al Aïn 2014). As discussed below, patterns of parent-offspring interactions depend on both the developmental status of the neonate and the litter size, as well as the breeding system of the mother (Clutton-Brock 1991; Nowak et al. 2000).

### Mother-Offspring Recognition

Discriminative care of offspring is common in mammals due to the high cost of lactation and other maternal behaviours (Clutton-Brock 1991; Gubernick and Klopfer 1981). Chemical cues are important in offspring recognition in many mammalian species including domestic ungulates (reviewed in Nowak et al. 2000; Poindron et al. 2007), rodents (Beach and Jaynes 1956; Ostermeyer and Elwood 1983; Porter et al. 1973), bats (Fanis and Jones 1996; Gustin and McCracken 1987) and pinnipeds (Pitcher et al. 2010a). One of the best studied examples is the domestic sheep, *Ovis aries*. Ewes can identify their own young shortly after parturition and prevent alien young from suckling (Keller et al. 2003; Poindron et al. 1993). Maternal behaviours and offspring recognition are disrupted following damage to the ewe’s olfactory system (Baldwin and Shillito 1974; Lévy et al. 1995; Morgan et al. 1975) or by restricting the ewe’s access to their lamb’s olfactory cues (Poindron and Neindre 1980; Poindron et al. 2007), indicating that chemical cues are important in identification. Offspring identification is based on a lamb’s individual olfactory signature, which is encoded by the genome (Porter et al. 1991; Romeyer et al. 1993). Although olfaction is clearly important in early offspring recognition, ewes can also recognise their lambs through auditory or visual cues (Alexander 1977; Ferreira et al. 2000; Terrazas et al. 1999). The use of multisensory signals to identify young also occurs in other species, including Mexican bats (Balcombe 1990; Gustin and McCracken 1987), laboratory mice (Cohen et al. 2011), Norway rats (Farrell and Alberts 2002) and Australian sea lions (Pitcher et al. 2010a; Pitcher et al. 2010b). Visual and auditory cues may allow recognition at a distance while olfactory cues provide confirmation at close quarters prior to nursing. However, exactly how such multisensory cues are integrated is unclear and is an important area of future study.

Identification of offspring may be particularly important in precocial species that breed in large herds or colonies, such that offspring from multiple females are present (see examples above). Here, females need to discriminate own from alien young to ensure that the benefits of parental care and lactation are received by own offspring. By contrast, in many altricial species, offspring are confined to a single den or nest until weaning (Gubernick and Klopfer 1981). As mothers are unlikely to encounter unrelated young within their nest, recognition of own young may be less important (Holmes and Sherman 1982). A recent study in domestic cats (*Felis silvestris catus*) found that despite being able to discriminate between the odours of own and alien young, females retrieved both equally (Banszegi et al. 2017). Solitary species, such as cats, may encounter unrelated young so infrequently that there is little need to discriminate them from own offspring. Further, the price of mistakenly rejecting own offspring, for example because they have picked up alien odours from the environment, may outweigh the cost of accepting unrelated young.

Recognition of young is expected to be particularly important for animals that raise their young in a communal nest. However, in many communally nesting species females nurse all young in the nest indiscriminately (degus: Ebensperger et al. 2006; mice: König 1989a; König 1989b; general review: Packer et al. 1992; evening bat: Watkins and Shump 1981), although some species may preferentially suckle own young during early lactation (Watkins and Shump 1981). To investigate the relative investment of communally nesting females, a study of degus (*Octon degus*) used a radionuclide to track milk transfer from mother to young in communal nests (Jesseau et al. 2009). Unrelated pairs of females preferentially nursed own offspring when sampled at 2 weeks old (though still gave some milk to their co-nesting partner’s offspring), whereas sister pairs nursed all young equally; there was no evidence of discrimination close to weaning at 4 weeks (Jesseau et al. 2009). Female degus can discriminate between body odours from own pups, sister’s pups and unrelated pups (Jesseau et al. 2008) and may use this discriminatory ability to preferentially nurse related offspring.

The ability to recognise own mothers by offspring may also be important in mammals as unfamiliar conspecifics often behave aggressively towards alien young. Although recognition of mothers has been demonstrated in some species, few studies have directly investigated the role of olfaction in recognition (Lickliter and Heron 1984; Poindron et al. 1993; Val-Laillet and Nowak 2008). A study in newborn lambs concluded that auditory and visual cues are more important than olfactory signals in maternal recognition (Nowak 1991). However, lambs were prevented from directly interacting with ewes during the experiment so it is not known whether odours might still facilitate maternal recognition in sheep at close contact.

Olfactory cues are also important in mother-offspring recognition in humans. Mothers can identify their own infant through odour alone (Kaitz et al. 1987; Porter et al. 1983; Schaal et al. 1980). These individually recognisable odours are produced by the infant rather than being deposited on the infant by the mother (Kaitz et al. 1987; Russell et al. 1983) and extensive postnatal interactions are not necessary for recognition to occur (Porter et al. 1983). Similarly, human babies can discriminate between odours emanating from their mother or an unrelated female (Cernoch and Porter 1985; Macfarlane 1975; Schaal et al. 1980). This discrimination occurred in breast and not bottle fed infants, suggesting odour recognition may be a learnt response (Cernoch and Porter 1985). Further support for olfactory learning in infants comes from a study by Schleidt and Genzel (1990), who showed that infants preferred their mother's perfume when lactating mothers perfumed their breasts.

### Olfactory-Mediated Suckling Behaviour

Evidence from several mammalian species shows that females release highly specific chemical cues to guide their young to the nipples and elicit suckling behaviour (Arteaga et al. 2013; Schaal and Al Aïn 2014). The best studied example is the European rabbit which releases a mammary pheromone that causes suckling behaviour in their pups (Schaal et al. 2003). This may be a particularly important adaptation in rabbits, which only nurse their pups once a day for approximately 5 minutes (Zarrow et al. 1965). During this time the pups, which are born blind, have to orientate to the female’s abdomen, locate and attach to the nipples and suckle efficiently, in the context of severe sibling competition (Bautista et al. 2005; Drummond et al. 2000). A series of elegant experiments demonstrated that the volatile component of rabbit milk, 2-methylbut-2-enal (2MB2), was responsible for the stereotyped searching-grasping behaviour typically seen in new-born pups (Schaal et al. 2003). 2MB2 acts as a pheromone as it elicits a species specific response (Schaal et al. 2003) and the behavioural activity is independent of learning (Schaal et al. 2003). Further, the releasing effect of 2MB2 is concentration dependent (Coureaud et al. 2004). In rabbits, as with many mammals, the growth and survival of pups is highly dependent on their ability to effectively suckle during the first few days (Coureaud et al. 2000). By increasing neonatal suckling success, 2MB2 increases offspring survival and female reproductive fitness. As well as producing a stereotyped behavioural response, 2MB2 also induces neonatal odour learning (Coureaud et al. 2010). When new-born rabbits are exposed to a mixture of 2MB2 and a neutral odorant (or mixture of odorants, see (Coureaud et al. 2008)), they exhibit a strong search-grasping response towards the neutral odorant alone 24 hours later (Coureaud et al. 2006). This learned response is evident after a single conditioning session and persists for five days after the initial exposure. Pheromone induced learning of odorants may facilitate improved orientation to the dam and localization of the nipple (Coureaud et al. 2010). Further, learning of odours in the nest may allow social recognition of familiar conspecifics, such as the mother or sibling nestmates, upon weaning. Male rabbits tend to avoid conspecifics scented with an odorant they learnt through association with 2MB2 as a neonate, suggesting that one function of neonatal olfactory learning may be to avoid inbreeding later in life (Coureaud et al. 2010).

Similar nipple searching behaviour is seen in other altricial species, although the mechanisms have not been investigated as thoroughly as in rabbits (Schaal and Al Aïn 2014). Murine rodents are attracted to the scent of a lactating female but washing of the nipple removes the searching behaviour seen in pups (Logan et al. 2012; Teicher and Blass 1976). Application of maternal amniotic fluid, saliva or milk restored suckling behaviour in laboratory mice (Logan et al. 2012). However, the same study showed that only amniotic fluid initiated suckling behaviour in pups delivered by caesarean section, suggesting that pups require prior exposure to learn maternal cues (Logan et al. 2012). The authors suggest that suckling behaviour in mice may be initiated by a learned signature odour, similar to those underlying maternal recognition in sheep (Logan et al. 2012). However, under different experimental conditions, Al Aïn et al. (2014) found that attraction to milk and colostrum odours by newborn mice did not require prior exposure. These studies highlight the difficulty in identifying mammalian pheromones and the extent to which different experimental conditions can impact results.

Odours are also important in initiating suckling behaviour in humans. Odour emitted from the breast of lactating females are attractive to newborns (Makin and Porter 1989). Breast odours regulate arousal states (Doucet et al. 2007) and elicit head turning (Makin and Porter 1989; Marlier and Schaal 2005), eye opening (Doucet et al. 2007), oral repsonses (Marlier and Schaal 2005) and crawling (Varendi and Porter 2001) in human newborn babies. The compounds underpinning these behavioural responses in newborns have yet to be identified but are likely to be present in the colostrum, milk or in secretions from specialised Montgomery glands situated in the areola surrounding the nipple (Doucet et al. 2009; Marlier and Schaal 2005; Mizuno and Ueda 2004).

## Conclusions

This review highlights the diversity of scent signals produced by female mammals, and the wide range of functions that these fulfil, including competitive signalling, sexual advertisement and facilitation of maternal behaviours. However, female chemical communication still remains poorly understood in the majority of mammals. We suggest several key areas for future study. First, although female scents can communicate a wealth of information about an individual, such as species, sex, reproductive state, health, dominance status and genotype (Brown and Macdonald 1985), the specific compounds or mixtures that signal such information have been identified in very few cases. Developments in molecular techniques have improved both the detection and identification of specific compounds in female scents. However, determination of the functional significance of such compounds, through a combination of behavioural testing and scent manipulation, is an essential requirement. Secondly, most research on chemical signalling in mammals has focused on domesticated and laboratory animals. While providing invaluable insight into both the functions and mechanisms of female chemical signalling, these animals may not necessarily be representative of most wild species. Thirdly, the majority of studies that have investigated scent communication in wild mammals have focused on rodent or primate species. Similarly, many of the functions discussed in this review have been investigated mainly in species with similar social structures; for example, studies investigating female signals of quality and male preference have focused mostly on species where males contribute to parental care. Broader investigation of female chemical signalling across a range of species with different social structures, breeding systems and ecological conditions are needed to explore the factors that influence the evolution of female chemical signals. Finally, understanding how chemical signals integrate with other sensory systems, such as auditory and visual signals, is a major challenge for future research.
